# Paroxysmal nocturnal haemoglobinuria in pregnancy–a systematic review with meta analysis

**DOI:** 10.1007/s00277-025-06353-7

**Published:** 2025-04-17

**Authors:** James E. Manning, Etienne Ciantar, Morag Griffin, Richard J. Kelly

**Affiliations:** 1 Centre for Haematology, Imperial College London, London, UK; 2 Department of Haematology, Imperial College Healthcare NHS Trust, London, UK; 3 Department of Obstetrics, Leeds Teaching Hospitals NHS Trust, Leeds, UK; 4 Department of Haematology, Leeds Teaching Hospitals NHS Trust, Leeds, UK

**Keywords:** Paroxysmal nocturnal haemoglobinuria, Pnh, Eculizumab, Pregnancy

## Abstract

The co-occurrence of Paroxysmal nocturnal haemoglobinuria (PNH) and pregnancy has traditionally been associated with poor feto-maternal outcomes. Eculizumab, a terminal complement inhibitor, is often used in pregnant patients, but the drug is not universally available. We therefore present the first systematic review with meta-analysis of outcomes in 190 pregnancies from 135 PNH patients, comparing those receiving eculizumab and those not. We searched PubMed for studies reporting on pregnancy outcomes in PNH. Thirty-five papers were selected for inclusion, the majority of which were case studies or small series. Eculizumab was used in 131 pregnancies. There was one maternal death reported in a woman who did not receive eculizumab. A higher rate of fetal survival was observed in pregnancies where eculizumab was used (82%) than those when it was not (69%). Miscarriage (< 24 weeks gestation) occurred twice as often in women not receiving eculizumab. Intrauterine death (≥24 weeks gestation), maternal thrombosis, bleeding (both antepartum and postpartum), and pre-eclampsia were over-represented in PNH pregnancies but did not significantly differ between treatment groups. Premature birth (< 37 weeks’ gestation) was observed in 32% of eculizumab pregnancies and 44% of non-eculizumab pregnancies. We conclude that Eculizumab appears to be safe and benefits pregnant women with PNH.

**Supplementary Information:**

The online version contains supplementary material available at 10.1007/s00277-025-06353-7.

## Introduction

Paroxysmal nocturnal haemoglobinuria (PNH) is a rare, acquired, clonal disorder of haematopoietic stem cells, characterised by complement-mediated intravascular haemolysis, and thrombosis [1]. Somatic loss-of-function mutations in the *PIGA* gene result in arrest of glycophosphatidylinositol (GPI) anchor synthesis. Absence of GPI-anchored complement inhibitory proteins CD55 and CD59 renders red cells exquisitely sensitive to intravascular lysis, resulting in a severe haemolytic phenotype [2]. Activation of coagulation, in part mediated by accelerated terminal complement activity leads to thrombosis [3–5]. PNH may occur *de novo* or on a background of aplastic anaemia or hypoplastic myelodysplastic syndrome, possibly due to clonal selection of GPI-deficient HSCs [6]. 

Pregnancy exacerbates the clinical phenotype of PNH, increasing maternal and fetal morbidity and mortality. Pregnancy is well documented as a prothrombotic state, additive to the risk observed in PNH, and early gestational haemodilution worsens anaemia [7, 8]. Classically a maternal death rate of 8% has been cited, with similarly poor fetal outcomes including high rates of fetal loss, prematurity and near ubiquitous low birth weights [9, 10]. It is for these reasons that pregnancy was traditionally discouraged in women with PNH.

Eculizumab, a terminal complement inhibitor and the first targeted therapeutic for PNH was approved in 2007 by the Food and Drugs Administration (FDA) and European Medicines Agency (EMA), revolutionising outcomes for non-pregnant patients, although it remains unavailable in a high proportion of countries worldwide [11]. Eculizumab treatment has been employed in pregnancy and limited data suggests that it confers benefits for pregnant women, with a favourable safety profile [12–14]. 

To date there is no systematic review of outcomes in pregnancies complicated by PNH, so there remains a need to provide further evidence to clinicians and patients to assist them in shared decision making around eculizumab therapy during pregnancy. We therefore present the first systematic review with meta-analysis of pregnancy management and outcomes in women with PNH.

## Methods

**Fig. 1:**
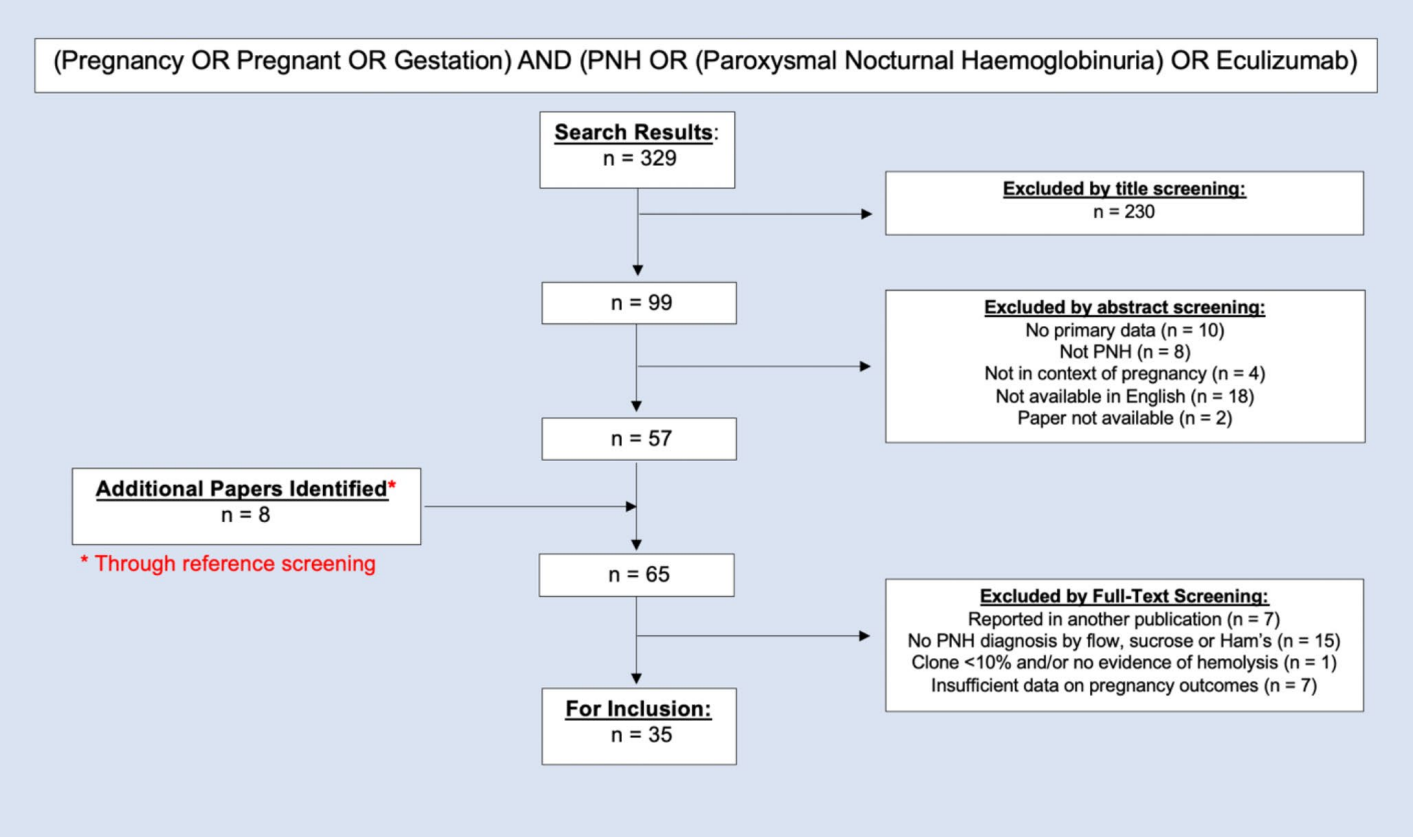
Database search strategy and article screening protocol

PubMed (Medline) was searched in February 2022 for studies reporting primary data on pregnancy in the context of classical haemolytic PNH (Fig. 1).

We initially wanted to restrict inclusion to only those patients with hemolytic PNH phenotypes, however there was inconsistent reporting in the published literature of key data such as lactate dehydrogenase levels, haemoglobin trends and presence or absence of haemoglobinuria at presentation, thus making this challenging to achieve. Given that haemolytic phenotypes typically appear when PNH clones are in excess of 10% in the peripheral blood, we pragmatically defined PNH cases as patients who met any one of the following criteria: flow cytometry with PNH granulocyte clone size of 10% or greater, a positive acid Ham’s or positive sucrose lysis test. Papers were excluded where: PNH diagnosis was not based on these modalities, where primary data was not reported, where there was insufficient data for meaningful analysis, or where cases were duplicated elsewhere in the literature. The search included non-English language papers, which were translated if they included more than one patient.

Two authors independently screened articles for inclusion, with a third opinion sought where there was disagreement. References of the articles selected for inclusion, and of review articles were screened to identify further papers for inclusion.

Data was collected on patient demographics, past haematological/obstetric history, and pregnancy outcomes. Breakthrough haemolysis was identified as a need for up-titration of eculizumab dose. Miscarriage and intrauterine death (IUD) were defined as fetal loss before or after 24 weeks gestation respectively. Prematurity was defined as birth before 37 weeks’ gestation. Intrauterine growth restriction (IUGR) was defined as a fetus falling through growth chart centiles, or a small for gestational age fetus with abnormal umbilical artery dopplers. Pre-eclampsia was identified as hypertension and proteinuria after 20 weeks’ gestation, as per standard diagnostic criteria [15]. 

Nominal data are presented as numbers and percentages, with odds ratios and 95% confidence intervals. For continuous data normality was assumed as per the central limit theorem if *n* > 30. In smaller samples, normality was assessed using Q-Q plots and the Kolmogorov-Smirnov test, with a *p* value of < 0.05 signifying non-normality. Parametric data was summarised using mean and standard deviation, with students t-test used to assess for difference. Non-parametric data were presented as median and ranges. Statistical significance was reached if *p* < 0.05.

## Results

Thirty-five papers were included in analysis, reporting on 190 pregnancies in 135 women, including five twin pregnancies (Supplementary Table 1) [9, 12, 13, 14, 16–45]. The majority of cases (*n* = 157 pregnancies) were published between 2011 and 2021, with a smaller number of more historical cases (published 1968–2010). Papers reporting data on women who received eculizumab were published between 2009 and 2022. Papers reporting data on women who did not receive eculizumab were published between 1968 and 2022, with half published after eculizumab gained regulatory approval (2007–2021). Most papers included were small case series or single case studies. There were two larger retrospective studies reporting on 45 and 75 pregnancies respectively [12, 14]. There was one small prospective study (*n* = 15) [13]. 

### Baseline characteristics of the women

80% of women received a diagnosis of PNH prior to their first documented pregnancy, with the remainder diagnosed during their index pregnancy or shortly following delivery. Data was reported for a single pregnancy in most women, with just under a quarter (*n* = 37) having two or more pregnancies.

Data on individual patient baseline characteristics including age, granulocyte clone size and past medical history was poorly reported, limiting meaningful analysis (Table 1). Of the available data, women who received eculizumab were older than those who did not (mean age 34 vs. 26, t(67) = 4.4, *p* = 0.0002). Individual PNH clone sizes were reported for only 30 women (15 who received eculizumab, 15 who did not), and based on this limited analysis were homogenous between groups (Table 1). One third of women had a history of bone marrow failure (aplastic anaemia or hypoplastic MDS). Around one in ten women had a history of venous thromboembolism, with no difference between those who went on to receive eculizumab and those who did not.

**Table 1:** Baseline characteristics of the women

	Eculizumab (*n* = 102)${^{\nabla }}$	Non-Eculizumab (*n* = 41)${^{\nabla }}$
**Presenting Features** ^T^		
Age at time of index pregnancy– mean (SD)*	34 (5.99)	26 (4.94)
Granulocyte clone size– median (range)^o^	80 (30–98)	81 (16–99)
Thrombosis history– n (%)	12 (12)	4 (10)
Bone marrow failure– n (%)^b^	34 (33)	16 (39)
**PNH Diagnosis**– n (%)		
Prior to index pregnancy	87 (85)	27 (66)
During index pregnancy	13 (13)	7 (17)
After index pregnancy	2 (2)	7 (17)

^T^– At time of index (first reported) pregnancy ^b^– Including aplastic anaemia and hypoplastic MDS

Data available for: ^*^n = 33 women receiving eculizumab, *n* = 35 not receiving eculizumab

^o^*n* = 15 women receiving eculizumab, *n* = 15 not receiving eculizumab

${^{\nabla }}$*n* = 8 women overlap both groups since they received eculizumab in one pregnancy but not a subsequent pregnancy (or vice versa)

### Pregnancy management

Eculizumab was used in 131 pregnancies (69%) and in two thirds of cases was commenced prior to conception. Three women discontinued eculizumab in early pregnancy, two enrolled in clinical trials, and the other because of fears of untoward effects on the pregnancy. All other patients continued eculizumab throughout pregnancy and the post-partum period. Of the 47 cases in which eculizumab was commenced during pregnancy, timing of initiation was available in only sixteen, with four women started in the first trimester, seven in the second and five in the third.

There were 59 pregnancies (31%) in which eculizumab was not used and half of these cases (*n* = 30) were published prior to the regulatory approval of eculizumab (pre-2007). In the remaining 29 cases, the reason for omitting eculizumab was not specifically documented. Granulocyte clone sizes were available in 24/29 of these cases with a median clone of 74.5% (range 16–99%), suggesting that a non-haemolytic phenotype was not the reason for withholding complement blockade.

There was variation in the management of thrombotic risk. Almost 90% of women on eculizumab received antenatal thromboprophylaxis, compared to only 50% of those who were not (Table 2). Median clone sizes were available in only a proportion of women and based on this limited analysis did not differ between treatment groups (*n* = 30, 24 anticoagulated, 6 not– 80.5% and 80.7% respectively). The most common agent was low molecular weight heparin (LMWH), with a small number of women receiving unfractionated heparin or synthetic heparinoid (Fondaparinux or Dextran). Treatment dose anticoagulation was employed in just under one third of cases. Data on the indication for treatment dose anticoagulation was reported in only 13 cases, 8 of which had a history of thrombosis, four of which did not, indicating the regimen was instituted prophylactically. In those who received thromboprophylaxis the universal practice was to commence this at the point of pregnancy diagnosis and continue throughout the antenatal period and up to six weeks’ postpartum.

Monitoring of anticoagulation was reported for only 27 pregnancies. Patients on LMWH (*n* = 23) were monitored according to peak anti-Xa levels in 22 cases, 21 of which were titrated to a target of 0.5-1 IU/mL, and one with a lower target of 0.3–0.5 IU/mL. In one case, LMWH was titrated according to D-dimer level, with anticoagulation intensified based on rising levels. The remaining patients (*n* = 4) were treated with UFH, one of which was on a continuous IV infusion, titrated according to APTT ratio. Three were on twice daily SC dosing, all of whom had D-dimer levels to guide dose changes. Of note, all cases in which D-Dimer was used to monitor anticoagulation were published by groups in Japan.

Overall, these data show that there remains significant variation worldwide in clinical practice as regards use of complement blockade and the use of anticoagulation. Where anticoagulation is used there is also variation in the timing of initiation, dose, agent, and monitoring.

**Table 2:** Management of PNH pregnancies

	Eculizumab	Non-Eculizumab
**Eculizumab Commencement**– *n* (%)		
* Prior to pregnancy*	84/131 (64.1)	-
* During pregnancy*	47/131 (35.9)	-
**Eculizumab Dose Titration– n (%)**		
* Standard Dose (900 mg/2 weekly)*	72/131 (55.0)	-
* Increased Dose*	59/131 (45.0)	-
* 1200 mg/2 weekly*	*13/59 (22.0)*	-
* 1500 mg/2 weekly*	*3/59 (5.1)*	-
* 1800 mg/2 weekly*	*6/59 (10.2)*	-
* 2400 mg/2 weekly*	*1/59 (1.7)*	-
* Dose unknown*	36/131 (27.5)	-
**Anticoagulation– n (%)**		
* Antenatal thromboprophylaxis*	116/131 (88.5)	30/131 (50.8)
* Agent used (of those anticoagulated)*		
* Low molecular weight heparin*	*113/116 (97.4)*	*21/30 (73)*
* Unfractionated heparin*	*2/116 (1.7)*	*8/30 (26.7)*
* Other*	*1/116 (0.9)*	*1/30 (3.3)*
* Dose*		
* Treatment dose*	*31/116 (27)*	*9/116 (30)*
* Intermediate dose*	*8/116 (6.9)*	*1/116 (3.3)*
* Prophylactic dose*	*76/116 (66)*	*19/116 (63)*

### Pregnancy outcomes

There was one maternal death in a woman who did not receive eculizumab. This patient, whose case was published in 1983, initially presented with both pancytopenia and evidence of haemolysis. She was found to have a hypocellular marrow on biopsy alongside a strongly positive sucrose lysis test at 68%. She therefore had concomitant haemolytic PNH and bone marrow failure (aplastic anaemia or hypoplastic MDS). During pregnancy she remained dependent on regular red cell and platelet transfusions. Her requirements for blood transfusion increased during pregnancy. Her pregnancy was complicated by severe haemolysis, biliary sepsis, and upper gastrointestinal haemorrhage. An IUD was diagnosed at 32 weeks’ gestation. She suffered a large pulmonary haemorrhage after delivery and died from septic shock just under 12 weeks postpartum.

Overall, there were live births in 78% of pregnancies, with a significantly higher rate of fetal survival in pregnancies in which eculizumab was used (82%) than those in which it was not (68%). This was largely mediated by a higher rate of spontaneous miscarriage in women who had not received eculizumab (Table 3). There were 13 episodes of miscarriage in women on eculizumab, which was started prior to pregnancy diagnosis in each case. This assocation remained statistically significant when we excluded papers published prior to 2007, when eculizumab gained regulatory approval, to correct for time of reporting as a confounder (OR 0.41, 95% CI 0.17–0.98, *p* = 0.046). There was no significant association between fetal survival and use of antenatal thromboprophylaxis (OR 0.52, 95% CI 0.23–1.21, *p* = 0.13). Elective termination occurred in three women not receiving eculizumab, and one who was. One woman who was receiving eculizumab required emergency salpingectomy for a ruptured right tubal ectopic pregnancy.

There were 14 IUDs with no significant difference in incidence between treatment groups. IUD followed the development of pre-eclampsia and IUGR in three cases, and placental abruption in two. Two women not receiving eculizumab had severe haemolytic crises in the third trimester, leading to fetal loss. There was one IUD at 33 weeks’ gestation in a dichorionic diamniotic twin pregnancy. Placental histopathology was available in five IUD cases, each demonstrating signs of placental insufficiency with thromboses, infarcts and fibrosis.

**Table 3:** Comparison of pregnancy outcomes between women receiving Eculizumab and those not

	Ecu *n* (%)	Non-Ecu *n* (%)	OR (95% CI)
**Pregnancy Outcomes**			
* Live birth*	112/136 (82.4)${^{\nabla }}$	40/59 (67.8)	-
* Fetal loss*	24/136 (17.6)${^{\nabla }}$	19/59 (32.2)	0.45 (0.2–0.9, *p* = 0.03)
* Miscarriage*	*13/136 (9.7)*	*11/59 (18.6)*	-
* Intrauterine death*	*9/136 (6.7)*	*5/59 (8.5)*	-
* Elective termination*	*1/136 (0.7)*	*3/59 (5.1)*	-
* Ectopic pregnancy*	*1/136 (0.7)*	*0/59 (0)*	-
**Maternal Complications**			
* Maternal death*	0/131 (0)	1/59 (1.7)	-
* Thrombosis*	7/131 (5.3)	5/59 (8.5)	0.61 (0.2–2.0)^NS^
* Haemorrhage*	20/131 (15.3)	7/59 (11.9)	1.34 (0.5–3.4)^NS^
* Pre-eclampsia* ^T^	10/110 (9.1)^T^	7/45 (15.6)^T^	0.54 (0.2–1.5)^NS^
Infection*	10/131 (7.6)	9/59 (15.3)	0.46 (0.2–1.2)^NS^
**Fetal Complications**			
* Intrauterine growth restriction* ^T^	9/110 (8.2)^T^	4/45 (8.9)^T^	0.95 (0.3–3.2)^NS^
* Structural anomalies*	1/136 (0.9)	3/40 (1.7)	-
**Mode of Delivery**			
* Vaginal*	58/112 (51.2)	26/40 (65)	0.62 (0.29–1.32)^NS^
* Spontaneous*	*35/58 (60.3)*	*17/26 (42.5)*	-
* Induced*	*23/58 (39.7)*	*9/26 (22.5)*	-
* Caesarean section*	54/112 (48.2)	14/40 (35.0)	-
* Elective*	*9/54 (8.0)*	*2/14 (14.3)*	-
* Emergency*	*11/54 (9.8)^*	*12/54 (92.3)^*	-
* Unknown*	*34/54 (30.4)*	*0*	-
Neonatal Outcomes			
Prematurity	36/112 (32.4)	18/40 (43.9)	0.61 (0.3–1.3)^NS^
Birth weight in g– mean (range)^0^	2713 (450–4290)	2570 (1700–3600)	-

${^{\nabla }}$ Including five twin pregnancies (*n* = 131 pregnancies, *n* = 136 fetuses, 1 IUD of a twin, 5 live births.)

Infection requiring antibiotics. Data available for *n* = 115 pregnancies

^T^ Expressed as percentage of pregnancies progressing beyond 20 weeks’ gestation (*n* = 155)

^o^ Data available for *n* = 25 neonates

^ Following failed induction of labour in *n* = 7 cases

### Maternal outcomes

Haemolysis was the most common maternal risk. Of women receiving eculizumab, 59 (45%) required up-titrations in their eculizumab dose to control breakthrough haemolysis. Data on final dosage required to control haemolysis was available in 23 cases (Table 2). Data on haemolytic markers and requirements for red cell transfusions were inconsistently reported making it difficult to comment on the risk of transfusion dependence and haemolysis between groups.

Thrombosis complicated just over 6% of pregnancies. No thromboses occurred in women receiving treatment dose anticoagulation. In women receiving eculizumab there were seven cases of thrombosis, all of which occurred in the early post-partum period with ongoing eculizumab treatment. There were five cases of splanchnic thrombosis, including two cases of Budd-Chiari syndrome (BCS), one lower limb deep vein thrombosis (DVT) and one pulmonary embolism. All women were on at least prophylactic dose LMWH, with one woman on intermediate dose anticoagulation. There were five thromboses in women not receiving eculizumab, four occurred antenatally and one occurred post-partum. Amongst these included two cases of BCS, one case of cerebral venous sinus thrombosis (CVST) at 37 weeks and a lower limb DVT at 15 weeks. One woman developed purpura fulminans type-DIC. Three of these women were receiving prophylactic LMWH at the time of diagnosis, two were not.

Bleeding was over twice as common as thrombosis, complicating around 14% of pregnancies. Nine women had minor, self-limiting bleeding episodes that did not require intervention (epistaxis, gum bleeding, minor haematoma). Two women suffered placental abruption leading to fetal loss. Primary post-partum haemorrhage (PPH) complicated 11 pregnancies, five of which required blood product support. There were two secondary PPH due to retained products of conception, which required surgical evacuation. One woman, who died in the post-partum period, suffered recurrent antenatal gastrointestinal haemorrhage and a post-partum pulmonary haemorrhage. One woman had an intracerebral bleed 12 days postpartum, and a further woman was diagnosed with purpura fulminans type DIC. Of the women identified with clinically significant bleeding episodes three were receiving therapeutic anticoagulation, eighteen prophylactic dose and six were not receiving any anticoagulation.

Pre-eclampsia was common, and although there was a trend towards decreased incidence of pre-eclampsia in pregnancies in which eculizumab was used (9.1%) than in those in which it was not (15.6%), this did not reach statistical significance. Nineteen pregnancies were complicated by infection requiring antibiotic treatment. There were no meningococcal infections in women receiving eculizumab.

### Fetal & neonatal outcomes

IUGR occurred in 13 cases and at similar rates between treatment groups. Congenital anomalies were detected in four fetuses, one exposed to eculizumab and three not.

Premature birth occurred in over one third of pregnancies that progressed to delivery. There was a trend towards a lower risk of premature birth in women who had received eculizumab (32% vs. 44%), but this did not reach statistical significance. Aetiology of premature birth was reported in 45/54 cases, of which obstetric factors were most common (*n* = 22). Haematological complications (*n* = 8) and elective delivery close to the point of fetal maturity (*n* = 9) accounted for the other cases. Spontaneous pre-term labour accounted for prematurity in only 13% of cases.

Twelve infants required prolonged hospital stays, eight due to prematurity, one due to meconium ileus secondary to Hirschsprung’s disease and one due to excessive primary weight loss. There were no neonatal deaths.

Eculizumab assays were performed on 23 cord blood and 14 breast milk samples. Of the cord blood samples eight (35%) were positive, with levels in the range of 11.1–21.2 µg/mL. Eculizumab was not detected in breast milk.

### Mode of delivery

Over half (55%) of infants were born by vaginal delivery. Induction of labour was required in 40% of those receiving eculizumab and 34% of those who did not. Indication for induction was reported for 22 pregnancies. In 11 cases induction was elected at the point of fetal maturity (37 weeks’ gestation), in eight cases because of obstetric complications and in a small number induction was required due to worsening anaemia or thrombocytopenia (*n* = 3).

Caesarean delivery occurred in 45% and 35% of women who received and who did not receive eculizumab respectively. Indication for caesarean delivery was documented in 31 cases, with 22 emergency and nine elective procedures. Indication for emergency caesarean delivery included fetal distress (*n* = 13), failed induction of labour (*n* = 7) and haematological complications (*n* = 2) including one case of severe anaemia and one of haemolytic disease of the fetus. Elective caesarean section was most often performed at the point of fetal maturity (*n* = 5), or due to persistent abnormal fetal presentation or lie (*n* = 3). In one case elective caesarean was indicated for maternal anaemia.

## Discussion

In this systematic review of pregnancy outcomes in women with PNH, we find that eculizumab use is associated with a lower rate of fetal loss, mediated by fewer spontaneous miscarriages in the first two trimesters. This remains statistically significant even when excluding papers published prior to the regulatory approval of eculizumab to correct for time of reporting as a confounder. Although fewer women in the non-eculizumab group received thromboprophylaxis, we found no association between fetal survival and the use of antenatal thromboprophylaxis. This suggests that at least some of the improvement in fetal survival is mediated by complement blockade rather than other aspects of management. Interestingly, women in the eculizumab group were significantly older, which would usually be associated with poorer outcomes. As such, it is possible that the true effect size of eculizumab on improving fetal survival is greater than demonstrated here.

Miscarriage occurred at a rate of just under 10% in women receiving eculizumab, similar to the rate in the general population and consistent with data from a recent publication of 15 pregnancies in PNH patients on eculizumab (miscarriage rate 7%) [46]. Intrauterine death complicates around 0.4% of pregnancies in the general population but is over-represented in PNH pregnancies. We therefore counsel women on the risk of late fetal loss and recommend regular fetal monitoring including ultrasound scans from 26 weeks’ gestation onwards to assess fetal growth, liquor volume and umbilical artery doppler studies to detect early placental insufficiency.

Pre-eclampsia has an incidence of approximately 1.5–7.7% in the general population depending on parity (higher in nulliparous women). These data therefore show that pre-eclampsia is over-represented in PNH patients [47]. This may relate to placental microvascular thrombosis and consequent shallow placental invasion [47]. The data presented here show a trend toward a reduced risk of pre-eclampsia in women treated with eculizumab, although this did not reach statistical significance. Aspirin prophylaxis (150 mg once daily) started at 12 weeks’ gestation and continued until 36 weeks’ gestation reduces the risk of pre-eclampsia and is standard of care for women at risk [47]. We therefore recommend our pregnant PNH patients take aspirin prophylaxis unless otherwise contraindicated. The development of hypertension, proteinuria or peripheral oedema during pregnancy is concerning for the development of pre-eclampsia and mandates urgent evaluation by an obstetrician.

We found that the management of thrombotic risk was very heterogeneous. Thrombosis rates were approximately equivalent between eculizumab and non-eculizumab pregnancies. Interestingly, there were no reported cases of thrombosis in women receiving treatment dose anticoagulation. This tentatively supports offering therapeutic anticoagulation for pregnant PNH patients, provided there are no contraindications. This approach is adopted by our own centre, where we typically anticoagulate women throughout pregnancy and the post-partum period with therapeutic doses of LMWH. Given the challenges of dosing LMWH in the context of gestational weight changes, we titrate LMWH to achieve peak Anti-Xa activity levels of 0.5-1.0 IU/mL.

Thrombosis prevention needs to be carefully balanced against the risk of bleeding, particularly given that aplastic anaemia and hypoplastic MDS frequently co-occur with PNH, both of which can elevate bleeding risk due to thrombocytopenia. In the data we present here, bleeding events were twice as common as thrombotic events and were associated with more intensive anticoagulation strategies. As such, a careful risk-benefit discussion should be had with each patient around anticoagulation, and women should be carefully monitored throughout pregnancy for bleeding.

Data on red cell transfusions was poorly reported between papers and therefore it was not possible to conduct a head-to-head comparison of transfusion requirements between the treatment groups. Increases in the dose of eculizumab to control ‘breakthrough’ haemolysis were required in just under half of pregnancies in which the drug was used, confirming findings published previously [12, 14]. 

The rate of congenital anomalies in our cohort (2%) is around the same as the UK average (2.2%) [48]. Only one infant who had been exposed to eculizumab had a detected congenital anomaly, suggesting that eculizumab is not teratogenic. The findings of low levels of eculizumab in cord blood are consistent with previous reports. Previous work has determined that although eculizumab can cross the placenta, it does so in levels too low to block complement activation in the neonate [31]. 

Limitations of this study include the relatively poor quality of available literature, high risk of bias of included studies and inconsistent reporting of some key data. For instance, clone sizes were available for only 30 women. In Kelly et al., (2015) median clone size was 63.7% (*n* = 61 women), lower than the 30 women for which individual data was available here [12]. We cannot exclude the possibility that heterogeneity in disease phenotype between patients may in part account for differences in clinical outcomes. However, we did restrict our inclusion criteria to patients with granulocyte clones > 10% or positive sucrose/acid hams tests in an attempt to assess a reasonably homogenous cohort of patients.

Data on neonatal outcomes was sparse and future study should seek to investigate neonatal outcomes in eculizumab exposed pregnancies in more detail. Furthermore, access to healthcare/obstetric care in countries with no access to eculizumab is likely to be variable and not entirely accounted for by the data reported here. Likewise, a higher proportion of women in the non-eculizumab group received their PNH diagnosis during pregnancy or after delivery, which is likely to contribute to the poorer outcomes observed in the non-eculizumab group. Despite this, these findings suggest that with high-quality, well-planned care for women with PNH, maternal and fetal outcomes can be generally good.

## Conclusions

Eculizumab appears to be safe for pregnant women with PNH as evidenced by a high rate of fetal survival and a trend toward reduced risk of premature birth and pre-eclampsia. The data tentatively support the routine use of treatment dose anticoagulation for thromboprophylaxis, although women should be carefully counselled on bleeding risk and close fetal monitoring instituted throughout pregnancy. Large high-quality prospective studies are required to validate these findings and provide more certainty to clinicians and patients on management decisions.

## Electronic supplementary material

Below is the link to the electronic supplementary material.

Supplementary Material 1

## Data Availability

Raw data extracted from individual papers included in the meta-analysis can be made available upon request to the corresponding author.
