# Supplementary material for: Paroxysmal nocturnal haemoglobinuria in pregnancy–a systematic review with meta analysis
Source: Ann Hematol. 2025 Apr 17;104(4):2517–25. doi: 10.1007/s00277-025-06353-7 (PMC12052813; doi:10.1007/s00277-025-06353-7)
Supplement: Supplementary file 1 — Supplementary Material 1 [file 277_2025_6353_MOESM1_ESM.docx]

| **Author** | **Year** | **Journal** | **Country of publication** | **Study design** | **Women (n)** | **Pregnancies (n)** |
| --- | --- | --- | --- | --- | --- | --- |
| Al-Dosari | 2021 | Saudi J Med Sci | Saudi Arabia | Case series | 4 | 9 |
| Alashkar | 2020 | Front Med (Lausanne) | Germany | Prospective | 9 | 16 |
| Allen | 2007 | Can J Anaesth | Canada | Case report | 1 | 1 |
| Aviles | 1984 | Ginecologa y Obstetrica de Mxico | Mexico | Case series | 4 | 4 |
| Bais | 1994 | Eur J Obstet Gynaecol Reprod Biol | Netherlands | Case report | 1 | 2 |
| Bastos | 2018 | Medicine (Baltimore) | Brazil | Case report | 1 | 1 |
| Bhattacharyya | 2000 | Med J Armed Forces India | India | Case report | 1 | 1 |
| Brown | 2002 | Nephrology | Australia | Case report | 1 | 2 |
| Danilov | 2009 | Leuk Res | USA | Case report | 1 | 2 |
| Fassett | 2021 | Case Rep Womens Health | USA | Case report | 1 | 2 |
| Fitzpatrick | 1991 | J Obstet Gynaecol | Republic of Ireland | Case report | 1 | 1 |
| Frakes | 1976 | Obstet Gynaecol | USA | Case report | 1 | 1 |
| Füreder | 2020 | Ann Haematol | Australia | Retrospective | 1 | 1 |
| Gessoni | 2015 | Blood Coagul Fibrinolysis | Italy | Case report | 1 | 1 |
| Greene | 1983 | Obstet Gynaecol Surv | USA | Case report | 1 | 1 |
| Hallstensen | 2015 | Immunobiology | Norway | Case series | 3 | 8 |
| Higgins | 2004 | J Obstet Gynaecol | Australia | Case report | 1 | 2 |
| Hurd | 1982 | Obstet Gynaecol | USA | Case report | 1 | 1 |
| Imai | 1989 | Arch Gynaecol Obstet | Japan | Case report | 1 | 1 |
| Kelly | 2015 | N Engl J Med | UK | Retrospective | 61 | 75 |
| Lauritsch-Hernandez | 2018 | Clin Case Rep | Switzerland | Case report | 1 | 2 |
| Li | 2018 | Clin Case Rep | Japan | Case report | 1 | 2 |
| Manning | 2022 | Obstetric Medicine | UK | Retrospective | 17^*^ | 24^*^ |
| Melo | 2011 | Blood Transfus | Portugal | Case series | 3 | 3 |
| Miyasaka | 2016 | Int J Haematol | Japan | Case series | 3 | 3 |
| Morita | 2013 | Int J Haematol | Japan | Case series | 2 | 2 |
| Nishimoto | 2018 | Haemodial int | Japan | Case report | 1 | 1 |
| Payne | 1968 | J Obstet Gynaecol Br Commonw | UK | Case report | 1 | 4 |
| Rodriguez-Ferreras | 2019 | J Reprod Infertil | Spain | Case report | 1 | 1 |
| Sasano | 2016 | Blood Coagul Fibrinolysis | Japan | Case report | 1 | 2 |
| Sharma | 2015 | Leuk Res Rep | USA | Case report | 1 | 1 |
| Singh | 2014 | Int J Reprod Contracept Obstet Gynaecol | India | Case report | 1 | 1 |
| Solal-Céligny | 1988 | Arch Intern Med | France | Case series | 4 | 9 |
| Vasbien | 2017 | Int J Lab Haematol | Belgium | Case report | 1 | 1 |
| Vekemans | 2015 | Blood Coagul Fibrinolysis | Belgium | Case report | 1 | 1 |

*n= 25 pregnancies duplicated within with Kelly et al., (2015) removed
